# Thermostability of Probiotics and Their **α**-Galactosidases and the Potential for Bean Products

**DOI:** 10.1155/2014/472723

**Published:** 2014-02-18

**Authors:** Xiaoli Liu, Claude P. Champagne, Byong H. Lee, Joyce I. Boye, Michel Casgrain

**Affiliations:** ^1^Institute of Agro-Product Processing, Jiangsu Academy of Agricultural Sciences, Nanjing 210014, China; ^2^Agriculture and Agri-Food Canada, Food Research and Development Centre, 3600 Casavant O. Saint-Hyacinthe, QC, Canada J2S 8E3; ^3^School of Biotechnology, Jiangnan University, Wuxi 214122, China; ^4^Bonduelle North America, 540, Chemin des Patriotes, Saint-Denis-sur-Richelieu, QC, Canada J0H 1K0

## Abstract

Soybeans and other pulses contain oligosaccharides which may cause intestinal disturbances such as flatulence. This study was undertaken to investigate **α**-galactosidase-producing probiotics added to frozen foods which can survive warming treatments used in thawing and consumption of the pulses. The maximum **α**-galactosidase activity (1.26 U/mg protein) was found in *Bifidobacterium breve* S46. *Lactobacillus casei* had the highest **α**-galactosidase thermostability among the various strains, with *D* values of 35, 29, and 9.3 minutes at 50°C, 55°C, and 60°C, respectively. The enzyme activity was less affected than viable cells by heating. However, the *D* values of two bacterial enzymes were lower than those of three commercial **α**-galactosidase-containing products. Freshly grown cells and their enzymes were more stable than the rehydrated cultures and their enzymes. *Practical Application.* Enzymes and cultures can be added to foods in order to enhance the digestibility of carbohydrates in the gastrointestinal tract. However since many foods are warmed, it is important that the thermostability of the enzymes be assessed. This paper provides data on the stability of **α**-galactosidase, which could potentially be added to food matrices containing stachyose or raffinose, such as beans.

## 1. Introduction

There is an increasing demand of nondairy applications of probiotic bacteria [1, 2] which indicate much interest by the manufacturers to use fruits and vegetables as carriers of probiotic bacteria. These data suggest that there is considerable global interest in the development and marketing of the vegetable-bean functional probiotic food.

Soybean and many other pulses are an excellent food source due to their high amounts of either dietary fibers, proteins, or micronutrients and phytochemicals [3, 4]. However, the consumption of soybean and legume products has been somewhat limited because of intestinal disturbances such as flatulence, which is caused by the presence of oligosaccharides. Oligosaccharides in beans, such as raffinose and stachyose, which are nondigestible and not assimilated in the small intestine by the human GI enzymes, are fermented by the microbiota and thus responsible for flatulence [5, 6]. Therefore, removal of oligosaccharides from beans could be an important factor in improving their consumption level [7].

Some studies have shown that adding probiotics to pulse products can indeed reduce gastrointestinal discomfort due to gas [5, 8, 9]. However, these studies were carried out in products which had fresh probiotic cultures and which had not undergone processing steps. Many processing, storage, and warming parameters are detrimental to cell viability and enzyme stability and, therefore, present a challenge in the development of probiotic or enzyme-containing legume products. Thus, heating at high temperatures such as those used in blanching, canning, or “stir-fry” cooking is unthinkable for probiotics because temperatures are above 80°C, which would kill the cells. However, food products are increasingly marketed frozen and are then warmed with microwave units before consumption [10, 11]. It is unknown to what extent probiotics or their *α*-galactosidases can survive warming temperatures which would reach up to 60°C.

The aim of the present study was to evaluate the effects of moderate heat treatments (simulating warming) on probiotic bacteria and their *α*-galactosidases, in order to ascertain the potential use of probiotics as suppliers of *α*-galactosidase in processed pulse-based foods.

## 2. Materials and Methods

### 2.1. Microorganisms and Culturing

Stock cultures of *Lactobacillus rhamnosus* 910, *Lactobacillus casei* CLbBCV_1_, and *Bifidobacterium breve* S46 were kept at −80°C in 20% glycerol. *Bifidobacterium longum* R0175 was obtained from Lallemand Health Solutions Inc. (Montréal, QC, Canada) in a freeze-dried form.

With the frozen strains, 1 mL stock cultures were thawed at room temperature and inoculated into 100 mL freshly sterilized MRS (Difco, USA) broth supplemented with 0.05% (w/v) L-cysteine hydrochloride (Cys). With the freeze-dried cultures, 1 g of free-cell powder was added into 9 mL rehydration medium (1.5 g peptone, 1 g tryptone, 0.5 g meat extract, 100 mL water) and incubated for 15 min at 37°C after vortexing for 10 sec. After diluting the cell suspension by adding 1 mL to 9 mL 0.1% (w/v) sterile peptone water, 1 mL diluted suspension was inoculated into 100 mL MRS-Cys broth. The cell suspension was then incubated at 37°C in an anaerobic incubator (85% N_2_, 10% H_2_, and 5% CO_2_) until pH between 4.3 and 4.5 was reached. The incubation time varied slightly between strains but was approximately 16 h.

### 2.2. Enzymes and Reagents

Three commercial sources of *α*-galactosidases were purchased from the market and analysed: Nutriteck Alpha-Galactosidase (Nutriteck, Chateauguay, QC, Canada), Beano (GlaxoSmithKline, Moon Township, PA, USA), and Digestive Advantage Gas Defense Formula (Ganeden Biotech, Mayfield Heights, OH, USA). *p*-Nitrophenyl-*α*-D-galactopyranoside (pNPG), *p*-nitrophenol, bovine serum albumin, and Bradford reagent kit were purchased from Sigma (Oakville, ON, Canada).

### 2.3. Enzyme Extraction

Bacterial cells were centrifuged at 7,000 g for 20 min at 4°C. After discarding the supernatant, the cell pellet was washed in 10 mL of 50 mM Na phosphate (pH 6.0) twice. Finally the cells were resuspended in 10 mL of the same buffer. The cells were incubated in an ice bath (4°C) and sonicated using a Misonix sonicator S4000 (Newtown, CT 06470, USA), with an output amplitude level of 6, and the total process time of 16 min was carried out in 4 cycles of 3 minutes on and 1 minute off. The cell debris was removed by centrifugation at 14,000 g for 20 min at 4°C. The supernatant was used as a crude enzyme extract.

### 2.4. Enzyme Assay

*α*-Galactosidases activity was assayed according to the modified method of Donkor et al. [12]. 50 *μ*L enzyme extract was mixed with 150 *μ*L 2% (w/v) *p*NPG and incubated at 37°C for 20 min. The reaction was stopped by addition of 200 *μ*L 0.1 mol L^−1^ sodium carbonate solution. The amount of *p*-nitrophenol released was measured with a spectrophotometer at 420 nm. One unit of enzyme activity was defined as the amount of enzyme that released one *μ*mol *p*-nitrophenol per min under the assay conditions.

### 2.5. Determination of Protein Concentration

An aliquot (0.1 mL) of the crude enzyme and 3 mL of the Bradford reagent were vortexed for 5 sec to mix thoroughly and incubated at room temperature for 30 min, followed by absorbance measurement at 595 nm. Bovine serum albumin (Sigma) was used to prepare the standard curve.

### 2.6. Effect of Temperature on Viability of Cell

Bacterial cultures in MRS were incubated using an Eppendorf Mastercycler PCR system (Eppendorf Scientific, Westbury, NY, USA) with high heating speed set at different temperatures in the range of 50–60°C. Cultures (0.15 mL) were placed in Eppendorf tubes, removed at specific time points, and immediately cooled in an ice bath, and then aliquots of 0.1 mL cell suspension were diluted with 9.9 mL of 0.1% sterile peptone water. In the first dilution tube we carried out a high-shear homogenization step with an Omni TH unit (Marietta GA, USA) equipped with Omni Tip plastic probe at 27 000 RPM for 30 sec. The subsequent dilutions were carried out in 0.1% sterile peptone water with blending with a vortex unit for 5 sec. The dilutions were plated on MRS agar and incubated at 37°C in an anaerobic incubator (85% N_2_, 10% H_2_, and 5% CO_2_). The *D* values (heating time required to generate a 1 log reduction on viable counts) were determined for each temperature and served to calculate the *Z* value (increase in temperature required to reduce the *D* value by a factor of ten).

In one series of assays with *B. longum* R0175, the cell pellet obtained after centrifugation was resuspended in 50 mM Na phosphate (pH 6.0) and this cell suspension was exposed to the 50–60°C thermal treatment instead of the pH 4.5 culture in the MRS broth. This treatment enabled examination of the effect of the medium, and particularly pH, on the thermostability of the cultures.

In another series of assays, instead of using the fresh liquid culture obtained after 16 h incubation, a *B. longum* R0175 cell suspension was treated at 50–60°C immediately after being rehydrated. The goal of this treatment was to evaluate the effect of the method of preparation of the cell suspension on thermostability.

### 2.7. Effect of Temperature on the Stability of Enzyme Preparation

Known units of enzyme solutions were prepared by rehydrating the commercial powders into 50 mM Na phosphate (pH 6.0) at room temperature. If necessary, further dilutions were carried out in 50 mM Na phosphate (pH 6.0). The thermostability assays were carried out by incubating the solutions at different temperatures in the range of 50–60°C. The same method and equipment as were used to evaluate the effect of heating on the CFU was applied to the enzymes samples. The activity of untreated enzyme was defined as 100%.

### 2.8. Statistical Analysis

All the experiments were carried out three times independently, in order to ascertain the reproducibility of the results. The data presented are the average of the three determinations. Paired *t*-tests and analysis of variance (ANOVA) were carried out with SigmaPlot software.

## 3. Results

### 3.1. *α*-Galactosidases Activity of Probiotic Strains

The *α*-galactosidases activities in probiotic strains grown in MRS are shown in Table 1. The organisms exhibited *α*-galactosidase activities at different degrees. *Bifidobacterium breve S46* had the highest *α*-galactosidase activity and biomass yield, but *Lb. rhamnosus* 910 showed the highest protein yield. Data show that enzyme yield depends on specific cell enzyme activity as well as biomass in the bioreactor.

### 3.2. Thermostability of the Bacterial Cultures

The *D* values between 50 and 60°C show that the overnight-grown lactobacilli cells in acidified MRS cultures are much more stable than the corresponding bifidobacteria cultures (Table 2). Assuming the product could be cooked or exposed to 60°C for 15 min before being eaten, it can be predicted that such a situation would result in a loss of approximately 95% of the viable cells. In the case of overheating of the product, *Lb. casei* appears to be the most appropriate culture because its *Z* value was higher than that of *Lb. rhamnosus*. The medium in which the cells are suspended during heating had a significant effect on the viability losses. Thus, when an overnight-grown *B. longum* R0175 culture in MRS at pH 4.5 was centrifuged and cells were resuspended in a pH 6.0 phosphate buffer, the *D* values were almost 10 times higher (Table 3) than those of the acid MRS (Table 2).

Under industrial conditions, as most food processors do not have fermentation vats designed to prepare fresh overnight cultures, they would add frozen or dried cells directly to the food matrix [13]. As a result, when inoculating a frozen food product, the cells would rehydrate during thawing and warming. Therefore, the stability of freshly rehydrated cells was compared to that of the overnight-grown cultures. Data show that the *D* values of *B. longum* R0175 were about 20% lower in the freshly rehydrated cultures as compared to liquid-overnight cultures (Table 3). Therefore, one could expect slightly lower stability in commercial products in which freeze-dried products are directly added.

### 3.3. Thermostability of the Enzymes

The commercial products had similar *Z* patterns for their *α*-Gal but showed significant differences with respect to *D* values (Table 4). The data provided by the suppliers state that the organism producing the *α*-Gal is *Aspergillus niger* which would normally result in similar results. The differences between Ganeden and other products might be due to the presence of a *Bacillus coagulans* probiotic in one of the products or to different *A. niger* strains. Also bulking and stabilizer agents added into each commercial enzyme may have affected the results.

There were much greater variations in the thermostability properties of the bacterial *α*-Gals than in the commercial ones. The *Z* values of the different probiotic strains were all statistically different (Table 4).

In similar phosphate buffer (pH 6.0) medium, *D* values of the bacterial enzymes (Table 4) were much higher than those of the viable counts (Table 3). The *D* values of three commercial *α*-Gal enzymes were higher than those of the probiotic bacteria (Table 4), presumably because of the fungal sources or spore-bearing *Bacillus* source of commercial products. Therefore, a greater quantity of *α*-Gal of probiotic bacteria would be needed than that of the commercial enzymes in order to obtain a given *α*-Gal activity after warming. The *Z* value of the *Lb. casei* enzyme was slightly higher than that of *Lb. rhamnosus* (Table 4). This suggests that *Lb. casei* would be a better choice than *Lb. rhamnosus* for food enrichment in the case of overheating.

The high stability of the commercial *α*-Gal was expected since Manzanares et al. [14] report a high *Aspergillus nigerα*-Gal activity at 50°C. There are also reports of high *α*-Gal activity at 50°C with *Lb. fermentum* [15]. It was noteworthy that two commercial products had very similar *D* patterns (Table 4). The Ganeden product contains *Bacillus* culture as well as a blend of enzymes, which included *α*-Gal. The commercial *α*-Gal is typically obtained from *Aspergillus niger*. These data suggest that the Ganeden activity appeared to be from the exogenous enzymes. Further studies are needed on the Ganeden product with respect to the potential contribution of the *Bacillus coagulans* culture to *α*-Gal activity.

## 4. Discussion

This study was conducted in a perspective that probiotics could be added to frozen foods, destined to be warmed in a microwave apparatus, which contain nondigestible oligosaccharides. The ultimate aim of enrichment with probiotics is to reduce gas discomforts following consumption. Preventing intestinal problems linked to low digestion of food carbohydrates has been achieved with yoghurt for a subpopulation that suffers from lactose maldigestion [16]. However, yoghurt is eaten directly and cells are not subjected to heating prior to consumption.

A sizeable portion of frozen foods are not stir-fried but are simply thawed in the bag using microwave units and served directly. In the latter, foods are preferably warmed and tasted at temperatures between 45 and 60°C [17–19], but the FDA Food Code specifies that 60°C or higher should be used [20]. As a result, thermostability of the bacteria and enzymes was estimated in the 50–60°C range in our study.

The inactivation temperature of *α*-galactosidases differed between strains and sources, and this was also noted in the literature. Viana et al. [21] prepared soybean *α*-galactosidases presenting maximal activity at 60°C, but the enzyme from *Talaromyces flavus* was inactivated after 5 h at 60°C [22]. Data from this study are in line with the latter [22].

Some legume-containing food blends may have an acid pH, particularly if tomatoes are added. Studies were therefore carried out on the thermostability of probiotics in an acid medium. It is well known that heat resistance of bacterial cells is reduced by low pH [23, 24]. Thus, as expected, *B. longum* R0175 cells in acidified MRS (pH 4.5) showed a thermal survival rate which was significantly lower than those that had been centrifuged and resuspended in a pH 6.0 PBS medium. These data suggest that manufacturers should preferably add probiotic cultures in a food matrix having a neutral pH when they are to be subsequently heated.

Preparing fresh probiotic cultures at a processing plant requires equipment and specialized personnel. Most food manufacturers might therefore prefer to inoculate concentrated dried cultures from specialized suppliers rather than to produce them at their own plant. Using dried cell cultures in the food industry has the added advantages of improved quality and reproducibility, as well as enhanced microbial safety and health benefits [25, 26]. However, during manufacturing, cellular damage occur during the freezing and drying processes [27–29]. As a result, many factors will influence viability assessment of bacterial cultures during rehydration and particularly the hydration matrix, temperature, and time [30]. Assays were therefore conducted to ascertain if freshly rehydrated cultures had different sensitivity to subsequent heating than overnight-grown liquid cultures. Data show that the *D* values of viable counts were lower in the rehydrated culture, but this was not statistically significant. Such was not the case, however, with the enzyme activity. The *α*-Gal of cells which have undergone one transfer (grown overnight) was less sensitive to heating than that of freshly rehydrated cells. It was not ascertained if this was due to sublethal damage the freeze-dried cultures possessed or if the fermentation process provided subsequent enhanced resistance to heat stress [31–33]. In light of the lower stability of the just-rehydrated culture, processors wishing to carry out direct inoculation in foods which are subsequently heated might find it useful to increase the inoculation level in order to compensate for the increased sensitivity of these cell suspensions upon subsequent warming.

In our study, *D* value and *Z* value were used to evaluate the thermostability of probiotic cells and enzymes. According to our results and based on their thermostabilities on viable counts, lactobacilli rather than bifidobacteria should be considered for future assays in heated food products. This is not necessarily the case for *α*-Gal activity, where stability of the *Bifidobacterium longum* enzyme was as high as that of the lactobacilli. Unfortunately, the thermostability of the *α*-Gal of the probiotics is still lower than that of commercial products. In order to increase the thermostability of the bacteria and their enzymes, studies on microencapsulation need to be carried out.

This study was a necessary “proof of concept” step to ascertain if some probiotics/enzymes could indeed survive a short heating period at 60°C. It also enabled us to select proper thermoresistant commercial probiotics/enzymes for further testing. Studies are currently under way to assess if the selected dried cultures blended with frozen beans can survive frozen storage (−20°C) and thawing in a microwave, as well as if there subsequently remains *α*-Gal activity in a simulated gastrointestinal system.

## Figures and Tables

**Table 1:** Growth and *α*-galactosidase activities of the probiotic strains.

Strain	Activity (U/mL)	Population (CFU/mL)	Protein (mg/mL)	Specific activity (U/mg protein)
*Lb. rhamnosus* 910	0.21	2.03 × 10^9^	4.58	0.04^a^
*Lb. casei* CLbBCV_1_	0.20	1.67 × 10^9^	3.82	0.04^a^
*B. breve* S46	2.20	8.50 × 10^9^	1.79	1.26^c^
*B. longum* R0175	0.48	1.64 × 10^9^	3.17	0.16^b^

^a,b,c^Values in a given column which are followed by the same letter are not statistically different (*P* > 0.05).

**Table 2:** Thermostability of overnight-grown microbial cultures as viable counts in acidified MRS (pH 4.5).

Strain	*D* value (minutes)			*Z* value (°C)
	50°C	55°C	60°C	
*Lb. rhamnosus* 910	81.0 ± 38.5^a^	29.1 ± 5.9^a^	5.8 ± 0.3^a^	9.2 ± 1.7^c^
*Lb. casei* CLbBCV1	34.9 ± 4.1^b^	29.2 ± 1.4^a^	9.3 ± 1.9^b^	17.4 ± 1.6^a^
*B. breve* S46	1.80 ± 0.06^c^	0.42 ± 0.0^b^	0.29 ± 0.01^c^	12.7 ± 0.1^b^
*B. longum* R0175	1.68 ± 0.22^c^	0.71 ± 0.01^b^	0.63 ± 0.03^c^	15.4 ± 0.75^ab^

^a,b,c^Values in a given column which are followed by the same letter are not statistically different (*P* > 0.05).

**Table 3:** Effect of culture preparation method on thermostability of *Bifidobacterium longum* R0175 cells and *α*-galactosidases in phosphate media at pH 6.0.

Preparation method	Biological material	*D* value (minutes)			*Z* value (°C)
		50°C	55°C	60°C	
Freshly rehydrated	Cell (CFU)	16 ± 2^a^	2.4 ± 0.1^a^	ND	5.1 ± 0.2^a^
	*α*-Galactosidases	263 ± 18^b^	97 ± 4^b^	52 ± 4^a^	16 ± 1^b^
Liquid-overnight culture	Cell (CFU)	19 ± 3^a^	2.8 ± 0.4^a^	ND	5.1 ± 0.5^a^
	*α*-Galactosidases	800 ± 100^c^	159 ± 10^b^	115 ± 1^b^	12 ± 1^b^

ND: not determined.

^a,b,c^Values in a given column which are followed by the same letter are not statistically different (*P* > 0.05).

**Table 4:** *D* and *Z* values of *α*-galactosidases from overnight-grown strains and commercial products.

Strain or commercial enzyme	*D* value (minutes)			*Z* value (°C)
	50°C	55°C	60°C	
Beano	2070 ± 289^a^	209 ± 2^a^	175 ± 14^a^	9.2 ± 0.7^cd^
Ganaden	1530 ± 241^b^	170 ± 9^b^	121 ± 6^b^	9.1 ± 0.6^cd^
Nutriteck	939 ± 53^c^	270 ± 25^c^	96 ± 1^c^	10.1 ± 0.2^bc^
*Lactobacillus rhamnosus *	1110 ± 101^c^	170 ± 12^b^	71 ± 5^d^	8.7 ± 0.6^d^
*Lactobacillus casei *	860 ± 81^c^	216 ± 10^a^	97 ± 7^c^	10.6 ± 0.8^b^
*Bifidobacterium breve *	1700 ± 287^b^	265 ± 31^c^	6.6 ± 0.4^e^	4.2 ± 0.2^e^
*Bifidobacterium longum *	800 ± 100^c^	159 ± 10^b^	115 ± 1^b^	12.0 ± 0.7^a^

^a,b,c^Values in a given column which are followed by the same letter are not statistically different (*P* > 0.05).
